# A density functional theory study of high-performance pre-lithiated MS_2_ (M = Mo, W, V) Monolayers as the Anode Material of Lithium Ion Batteries

**DOI:** 10.1038/s41598-020-63743-9

**Published:** 2020-04-23

**Authors:** Tingfeng Liu, Zhong Jin, Dong-Xin Liu, Chunmiao Du, Lu Wang, Haiping Lin, Youyong Li

**Affiliations:** 1 Institute of Functional Nano & Soft Materials (FUNSOM), Jiangsu Key Laboratory for Carbon-Based Functional Materials & Devices, Soochow University, 199 Ren’ai Road, Suzhou, 215123 Jiangsu P.R. China; 2 Computer Network Information Center, Chinese Academy of Sciences, Beijing, 100190 China; 3 Center of Scientific Computing Applications & Research, Chinese Academy of Sciences, Beijing, 100190 China; 4 Jinduicheng Molybdenum Co. Ltd., No. 88 Jinye 1st Road, High-tech Zone Xi’an, Shanxi, P.R. China

**Keywords:** Density functional theory, Materials for energy and catalysis

## Abstract

Recent experimental study shows that the pre-lithiated MoS_2_ monolayer exhibits an enhanced electrochemical performance, coulombic efficiency of which is 26% higher than the pristine MoS_2_ based anode. The underlying mechanism of such significant enhancement, however, has not yet been addressed. By means of density functional theory (DFT) calculations, we systematically investigated the adsorption and diffusion behavior of lithium (Li) atoms on the MS_2_ (M = Mo, W, V) monolayers. On the pre-lithiated MS_2_ monolayers, the adsorption energy of extra Li ions are not significantly changed, implying the feasibility of multilayer adsorption. Of importance, the Li diffusion barriers on pre-lithiated MS_2_ are negligibly small because of the charge accumulation between the diffusing Li ions and the pre-lithiating Li layer. Correspondingly, we report that the pre-lithiation should be a general treatment which can be employed on many transition-metal di-chalcogenides to improve their storage capacities and charge-discharge performance in Li ion batteries. In addition, we propose that the pre-lithiated VS_2_ may serve as an outstanding anode material in LIBs.

## Introduction

The Lithium-ion battery (LIB) has been regarded as one of the most indispensable and promising devices in the fields of telecommunications, electric automobiles and electric power grids^1,2^. Today, graphite is widely used as the anode material of commercial LIBs owing to its layered structure, good electric conductance and excellent chemical stabilities^3,4^. Nevertheless, the maximum specific capacity of lithium ions of graphite (LiC_6_) is only 372 mA∙h ∙ g^−1^. As a result, numerous researches have been devoted to the searches of new anode materials with higher energy densities^1,5–7^. In addition to the specific capacity, columbic efficiency has also been employed to evaluate the performance of electrodes in LIBs. Thus, an ideal anode material, should not only accommodate densely packed Li ions, but also allow for fast Li diffusions to promote the charge-discharge rate^1,8–10^. In the past decade, a number of two-dimensional (2D) materials, including transition-metal oxides, di-chalcogenides (MO_2_ and MS_2_) and BN, have been successfully synthesized^11–13^. Their electronics properties and potential applications in devices have also been explored and proposed as electrode material for LIBs^14–20^. Very recently, Yang *et al*. report that the coulombic efficiency of MoS_2_ can be significantly improved by the pre-lithiation treatment, in which the MoS_2_ is on direct contact with lithium foils^21^. Despite the improved performance of MoS_2_ upon pre-lithiation, the underlying mechanism however, has not yet been addressed. Herein, systematic Density Functional Theory (DFT) calculations have been conducted to explore (i) the chemical insights of the enhanced performance after pre-lithiathion, and (ii) the effect of pre-lithiathion on other MS_2_ nanosheets. Our results revealed that the pre-lithiathion allows for multilayer adsorption and fast diffusion of Li ions on the MS_2._ In addition, pre-lithiathion may serve as a general treatment for improving the performance of MS_2_ anode in LIB. Last but not least, the VS_2_ monolayer provides relatively high Li binding strength, negligibly small Li diffusion barriers, and large theoretical capacity comparing with MoS_2_ and WS_2_ counterparts. We thus propose that the pre-lithiated VS_2_ monolayer is an outstanding anode material for LIBs. These results may open up a new avenue for the development of the next-generation high-performance LIBs.

## Computational Details

The DFT calculations were carried out with the Vienna ab-initio Simulation Package (VASP) code^22–25^. The projector augmented-wave potentials (PAW)^26^ and the Perdew–Burke–Ernzerhof (PBE) generalized gradient approximation (GGA) functional^25,27^ were used to describe the electron–ion interactions and electronic exchange correlations, respectively. The effect of on-site Coulomb interactions on the binding of Li ions on the MoS_2_, MoSe_2_, WS_2_ and WSe_2_ have been investigated by previous theatrical studies^28^. It was shown that the binding energy, binding height and diffusion barriers of Li ions are not significantly affected by the on-site Coulomb interactions. Correspondingly, the PBE functional was selected in this work. The conjugate gradient scheme was used to relax all atomic positions and lattice constants until the components of the forces on each atom is of the order of 10^−3^ eV Å^−1^. A plane-wave basis set with kinetic energy cutoff is set as 500 eV to ensure the accuracy of the simulation results. The number of K-mesh was (16 × 16 × 1) for the primitive MS_2_ unit cell and scaled according to the size of the supercells in the total energy and self-consistent-field (SCF) potential calculations. Based on the primitive cell (1 × 1), different supercells including (2 × 1), (2 × 2), (3 × 3), and (4 × 4), hexagonal structures as the ideal models are used to analyze the adsorption of lithium. The corresponding Brillouin zones of the (2 × 1), (2 × 2), (3 × 3), and (4 × 4) supercells are sampled with the Γ-centered k-point grid of 9 × 9 × 1, 8 × 8 × 1, 6 × 6 × 1, and 2 × 2 × 1, respectively. The lattice constants of MoS_2_ (3.186 Å), WS_2_ (3.186 Å) and VS_2_ (3.236 Å) were obtained from our DFT calculations. These lattice constants are in good agreement with the experimental values^29–33^. A vacuum of 20 Å along the z-axis was applied to prevent interlayer interactions from transnationally periodic images. The Climbing Image Nudged Elastic Band (CI-NEB) method was used to find the saddle points and minimum energy paths between the initial and final states^34–36^.

## Results and Discussion

**Figure 1:**
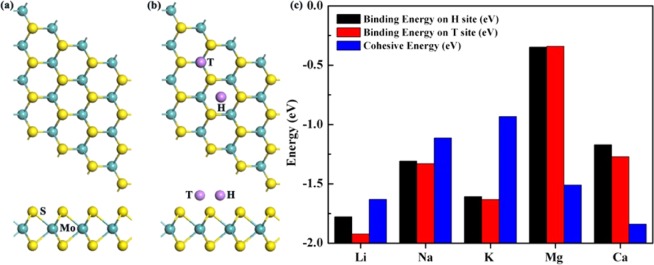
The top and side views of the optimized structures of (**a**) a MoS_2_ monolayer and (**b**) the top (T) and hollow (H) binding sites of a metal ion adsorbed on the MoS_2_ monolayer. The Mo atoms, S atoms and the binding sites are represented with green, yellow and purple circles, respectively. (**c**) The binding energies and metal cohesive energies of Li, Na, K, Mg, Ca on MoS_2_.

In two-dimensional transition-metal di-chalcogenides, the atomic layer of metal elements are sandwiched between two S layers. As shown in Fig. 1(a), the Mo−S bond length of the 2H-MoS_2_ is 2.41 Å, and the Mo−S − Mo bond angle is 80.68°, agreeing well the previous theoretical results^37^. Two binding sites are considered for analyzing the adsorption of Li ions on the MoS_2_. The top site (T site) is directly above one Mo atom, while the hollow site (H site) is above the center of a hexagon, as shown in Fig. 1(b). We have also examined the other possible adsorption sites (e.g. above the S atom), however, the adsorbed Li ion is observed to move to the neighboring T site after structural relaxation. The binding energy of metal atoms on the MS_2_ is defined as:

1 $${\text{E}}_{\text{b}}=({\text{E}}_{\text{nLi}-{\text{MS}}_{2}}-{\text{nE}}_{\text{Li}}-{\text{E}}_{{\text{MS}}_{2}})/\text{n}$$

The E_nLi-MS2_ is the total energy of the coupled structure, in which n Li ions adsorbing on the MS_2_. E_Li_ is the energy of an isolated Li atom in a vacuum. E_MS2_ is the energy of an isolated MS_2_ monolayer. And n is the number of adsorbed Li atoms. According to such definition, a more negative binding energy indicates a more favorable exothermic interaction between MS_2_ and Li atoms. As shown in Fig. 1, the adsorption of a Li ion at the T site (−1.94 eV) is more stable than that on the H site (−1.78 eV), with a Li-S distance being 2.37 Å, consisting well with previous theoretical studies^28,37^. In addition to Li ions, the adsorption of other metal elements which possess potential barrier applications have also been calculated. The binding energies of different adsorbing atoms and their corresponding cohesive energies are shown in Fig. 1(c). It can be seen that the binding of Li, Na and K atoms on MoS_2_ are stronger than the metallic bonds in their bulk structures. This suggests that the MoS_2_ may also be employed as anode materials for Na and K ion batteries.

**Figure 2:**
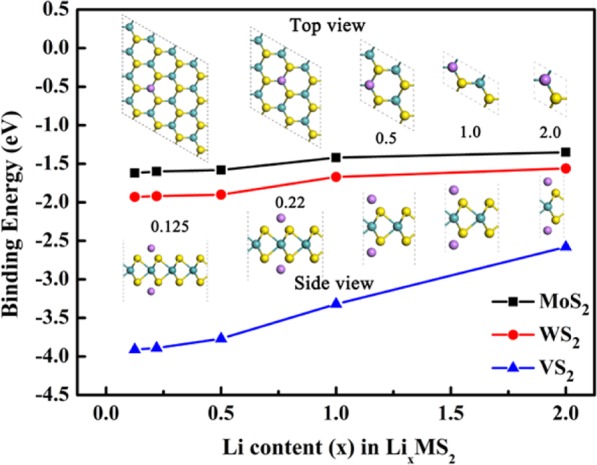
The top and side views of Li_x_MS_2_ and their averaged Li binding energies on MS_2_ monolayers.

Subsequently, the Li storage capacities of MS_2_ monolayer (M = Mo, W, V) were investigated. A series of Li/MS_2_ configurations with different stoichiometry of LixMS_2_ (x = 0.125, 0.222, 0.500, 1.000, and 2.000) were constructed by adding one Li ion on each side of the (4 × 4), (3 × 3), (2 × 2), (2 × 1) and (1 × 1) supercells, respectively. As shown in Fig. 2, the binding energies of Li ions decreases with increasing Li coverages. It is worthy to note that the Li binding energies on VS_2_ are much larger than on other MS_2_. When x = 2, full Li coverages are achieved on both sides of MS_2_. It is seen that the averaged binding energies of Li ions on fully covered VS_2_, MoS_2_ and WS_2_ are −2.58 eV, −1.35 eV and −1.56 eV, respectively. This indicates strong attractive interactions between Li ions and MS_2_ monolayers at the full coverage.

The Li_2_MS_2_ represents the highest Li storage capacity on bare MS_2_. At this coverage, the theoretical capacity can be calculated with the following equation:

2 $$\text{C}\,=\,cnF/{\text{M}}_{{\text{MS}}_{2}}$$

here c is the number of adsorbed cations on a MS_2_ unit and *n* is the valence state of fully ionized cations from electrolyte, *F* is the Faraday constant (26801 mA∙h∙mol^−1^), and ${\text{M}}_{{\text{MS}}_{2}}$ the molar weight of MS_2_. In this case, c is 2 at the full coverage, and n is 1for Li ions. Correspondingly, for the adsorption capacities are 334.87, 256.49 and 465 mA∙h ∙ g^−1^ for the pristine MoS_2_, WS_2_ and VS_2_ monolayers, respectively.

**Figure 3:**
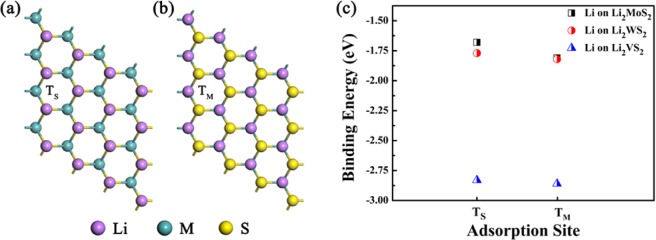
The top and side views of the pre-lithiated MS_2_ monolayer (M = Mo, W, V), with one Li atom adsorbing (**a**) above the S atoms (T_S_ site) and (**b**) above each metal atoms (T_M_ site). (**c**) The binding energies of a full coverage of Li atoms adsorbing on MS_2_ monolayers at the T_S_ and T_M_ sites.

Previous experimental studies show that the pre-lithiated MoS_2_ monolayer exhibit better performance compared with the pristine MoS_2_^21^. In order to obtain an in-depth understanding, the adsorption and diffusion of extra Li atoms on the pre-lithiated MS_2_ are investigated. Firstly, as shown in Fig. 3, two possible pre-lithiated configurations have been considered, the layered Li atoms prefer to adsorb above the T_M_ site of the MS_2_ monolayer with the binding energies being −1.81 eV (MoS_2_), −1.82 eV (WS_2_), and −2.86 eV (VS_2_), respectively. The corresponding Li-S distances are 2.45 Å (MoS_2_), 2.51 Å (WS_2_) and 2.32 Å (VS_2_).

**Figure 4:**
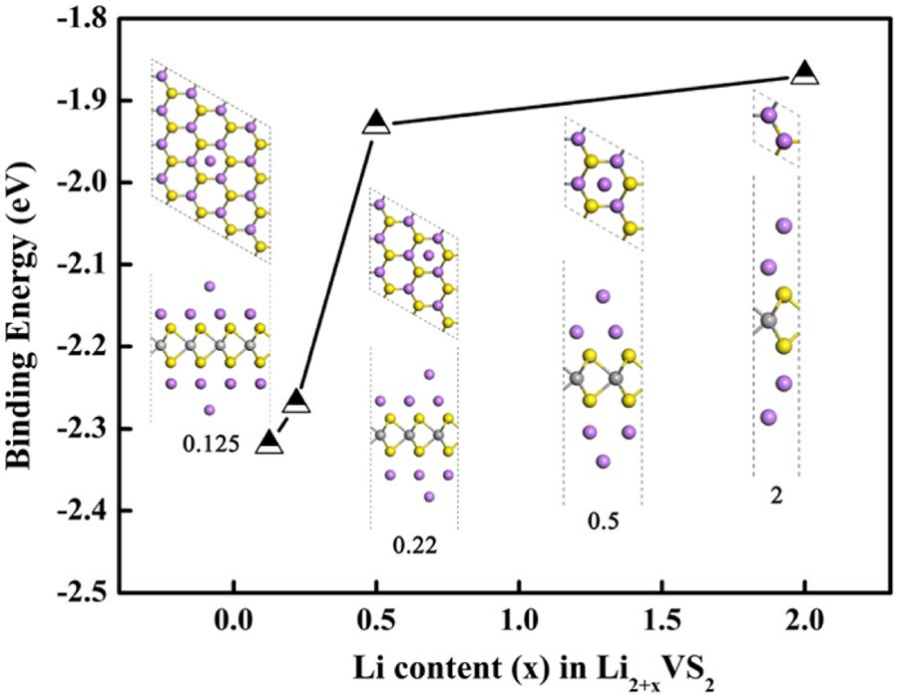
The trends of the binding energies of the Li ion adsorbing on the pre-lithiated VS_2_ (Li_2_VS_2_) with increasing Li coverages.

Figure 4 shows the configurations and corresponding binding energies of extra Li atoms adsorbing the pre-lithiated VS_2_ (Li_2_VS_2_) monolayer with various coverages. As seen, the binding energies of the Li ions on Li_2_VS_2_ monolayer decreases gradually with the elevation of the related storage ratio (x). On the pre-lithiated VS_2_, the Li ions used for pre-lithiation are assumed anchored on the VS_2_ and thus the Li storage capacity is defined as:

3 $$\text{C}=cnF/{\text{M}}_{{\text{Li}}_{2}{\text{MS}}_{2}}$$

maximum theoretical capacity of the Li atoms on the pre-lithiated MS_2_ (M = Mo, W, V) monolayers were 308.14, 204.70 and 415.67 mA∙h ∙ g^−1^ respectively. Thus, from the point of the binding energy and the theoretical capacity, Li_2_VS_2_ is relatively more suitable for LIBs anode materials for the higher binding energy and theoretical storage capacity.

**Figure 5:**
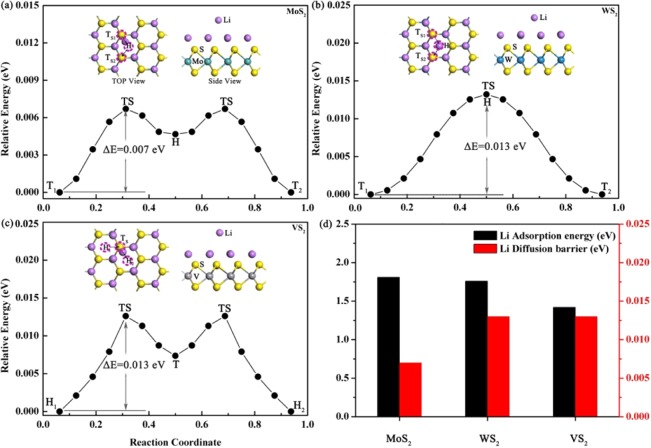
The energy profiles of Li diffusion on the pre-lithiated (**a**) MoS_2_, (**b**)WS_2_, and (**c**)VS_2_. (**d**) The Li binding energy at T site and diffusion barriers.

**Figure 6:**
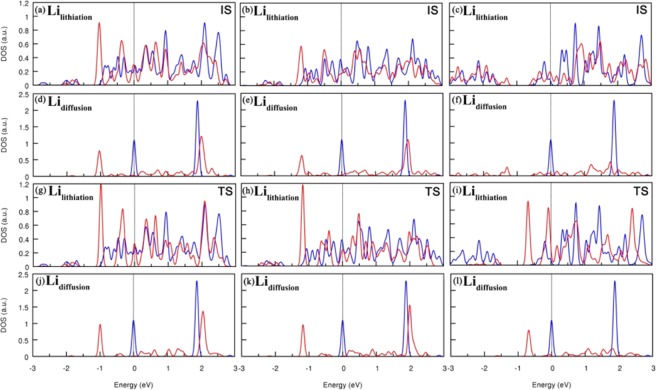
The corresponding local densities of states (LDOS) of the initial states (IS) and transition states (TS) of Li diffusion on Li_2_MS_2_ monolayers. (**a–c**) are the LDOS of the pre-lithiated Li atoms with (blue curve) and without (red curve) an additionally adsorbed Li atom. (**d–f**) are the LDOS of a Li atom in the vacuum (blue curve) and on the Li_2_MS_2_ surfaces (red curve) of the IS structures. (**g–i**) and **(j–l**) are the LDOS of corresponding Li ions of the transition states.

The performance of an electrode material is closely related the mobility of the adsorbed Li ions^38^. In general, a lower diffusion barrier means a higher diffusion rate^39,40^. Thus it is necessary to study the diffusion behavior Li ions when the Li_2_MS_2_ monolayers are used as the substrates. The migrations of the Li atom among the T site and the H site are studied using the CI-NEB method. The red circles and black arrows in Fig. 5 represent the diffusion pathway of the Li atom from the most stable adsorption site (T site or H site) to the next equivalent stable adsorption site. As seen in Fig. 5(a–c), when Li ions only need to overcome very small energy barriers to diffuse on the pre-lithiated MS_2_. Taking into account that the Li diffusion barriers in graphite (0.22 eV) and the pristine MS_2_ (0.22 eV) (M = Mo, W, V) are much higher than those on the he pre-lithiation MS_2_^3,28,37,41,42^, we can conclude that the pre-lithiation is an effective treatment for MS_2_ to achieve enhanced charge-discharge rates. The effect of Li diffusion barriers to the charge-discharge rates can be roughly estimated with the Arrhenius equation, D ∝ exp(−E_barrier_/k_B_T), where E_barrier_ and k_B_ are the Li diffusion barrier and Boltzmann constant. T is the temperature^43^. As can be seen, the diffusion constant increases exponentially with the decreasing diffusion barrier at a constant temperature. Please note that on the three Li_2_MS_2_ substrates, the pre-lihtiated VS_2_ monolayer is the most optimized anode material in terms of high Li binding energy and low diffusion barrier and the high Li adsorption capacity. Figure 6 summarizes the LDOS of the initial states (IS) and transition states (TS) of Li diffusion on Li_2_MS_2_ monolayers.

One of important factors for estimating the performance of LIB anode materials is the electric conductivity. Many pristine MS_2_ are semiconductors with large band gaps, implying poor electric conductivity^44–46^. As seen, all Li_2_MS_2_ monolayers are conducting materials. More detailed analysis of the LDOS plots shows that when a Li ion adsorbs on the Li_2_MS_2_ monolayer, the electronic states of Li ions are more hybridized, indicating that the interactions between the adsorbed Li and pre-lithiating Li layer are chiefly metallic bonding. This is consisted with the previous theoretical investigates^47^.

**Figure 7:**
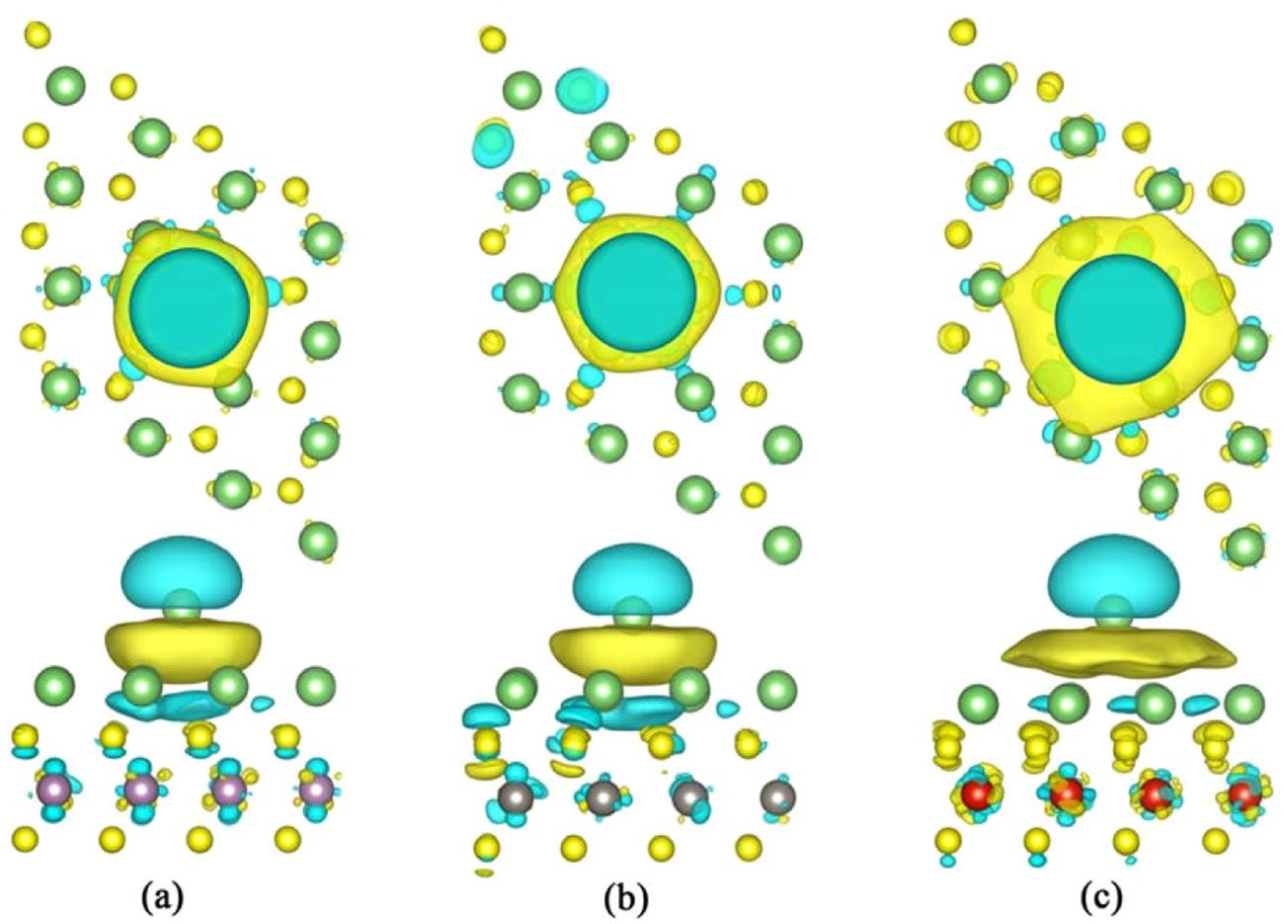
The top and side views of the differential charge densities of the transition states of the diffusing Li atom on the (**a**) Li_2_MoS_2_, (**b**) Li_2_WS_2_ and (**c**) Li_2_VS_2_ monolayers. The light blue and yellow contours (isosurface = 0.001 e/Å^3^) represent the charge deletion and charge aggregation, respectively.

The differential charge densities were calculated in order to identify the bonding characteristics between the diffusing Li ion and the Li_2_MS_2_ substrates. As clearly shown in Fig. 7, the electrons are accumulated between the diffusing Li ion and the Li_2_MS_2_. In addition, the areas of such charge accumulations expand on three neighing Li ions in the Li_2_MS_2_, indicating that the accumulated electrons are delocalized. This agree well with the PDOS analysis that the interactions are metallic bonding. As a result, the migration of the diffusing Li ion does not need to break the Li-Li_2_MS_2_ bonds. Correspondingly, the Li diffusion barrier on Li_2_MS_2_ should be very small, which is in good consistence with our CI-NEB calculations.

## Conclusions

In conclusion, the adsorption of Li ions on the surface of the pristine/pre-lithiated MS_2_ monolayer (Li_2_MS_2_, M = Mo, W, V) are systematically investigated. Our calculations showed that the optimal adsorption sites of Li ions on the pristine MS_2_ is the on-top site of the metal atoms. A pre-lithiating Li layer is formed when all the on-top sites are occupied by a Li ion. The pre-lithiation of MS_2_ (M = W and V) will enhance the adsorption and diffusion of Li ions. Although the Li binding energy on the clean MS_2_ and the pre-lithiation are not significantly different, the Li diffusion barriers on the pre-lithiated MS_2_ are much less than those on the clean MS_2_, implying a fast charge-discharge property. In particular, we report that the pre-lithiated VS_2_ is a very promising anode materials in the Li ion barriers, due to strong Li binding interactions and negligibly small Li diffusion barriers on the Li_2_VS_2_. Thus, this work not only interprets the in-depth working principles of the reported pre-lithiation for MoS_2_, but also propose that the pre-lithiated VS_2_ may serve as one of the best anode materials in LIBs.
